# MiR-34a suppresses amphiregulin and tumor metastatic potential of head and neck squamous cell carcinoma (HNSCC)

**DOI:** 10.18632/oncotarget.3148

**Published:** 2015-02-05

**Authors:** Jiali Zhang, Yu Wang, Xinming Chen, Yi Zhou, Fangyan Jiang, Jirong Chen, Li Wang, Wen-Feng Zhang

**Affiliations:** ^1^ The State Key Laboratory Breeding Base of Basic Science of Stomatology (Hubei_MOST) & Key Laboratory of Oral Biomedicine Ministry of Education, School & Hospital of Stomatology, Wuhan University, Wuhan, China; ^2^ Oral Histopathology Department, School and Hospital of Stomatology, Wuhan University, Wuhan, China

**Keywords:** Head and neck squamous cell carcinoma, miR-34a, amphiregulin, metastasis

## Abstract

MiR-34a is a well-known tumor metastasis inhibitor, but only a few target genes involved in metastasis have been identified. In HNSCC, the role of miR-34a in metastasis has not been fully elaborated, and the target gene of miR-34a is still blind. Here we addressed that, the relative lower expression of miR-34a is associated with HNSCC lymphatic metastasis. HNSCC metastasis was found to be strongly suppressed *in vitro* and *in vivo* by over-expressing miR-34a. In order to screen the possible target genes of miR-34a in HNSCC, a microarray-based differential mRNA profiling mediated by miR-34a over-expression was performed, and AREG was identified as a pivotal target. We demonstrated that the mRNA and protein levels of AREG were greatly reduced when forcing miR-34a expression. The correlation between AREG mRNA levels and HNSCC metastatic phenotype was also significant in HNSCC tissues (*p* < 0.01). Moreover, the results of luciferase assay provided the further evidence that miR-34a degraded AREG mRNA through targeting the 3′-UTR site. Restoration of AREG expression partially rescued miR-34a-mediated cell invasion defects *in vivo* and *in vitro*. Additionally, Over-expressing miR-34a greatly reduced EGFR and uPA, which were reversed by re-expression of AREG. Taken together, these findings indicate that miR-34a targets AREG, and is essential in inhibition of HNSCC metastasis.

## INTRODUCTION

Head and neck squamous cell carcinoma (HNSCC) is an invasive epithelial neoplasm with a propensity to early and extensive lymph node metastases [1]. Epidemiological statistics show that HNSCC represents the sixth most common lethal malignancy by incidence which affects approximately 650,000 people worldwide annually and leads to above 350,000 deaths [2]. Because of the multi-factorial etiopathogenesis of HNSCC, the development of multimodality treatments is suboptimal in recurrent and/or metastatic disease and only half of the patients are alive in 5 years [3, 4]. Despite of the advances in primary HNSCC treatment, the lack of good molecular targets and biological heterogeneity hinder the development of gene therapy in metastatic HNSCC [4, 5]. Therefore the investigation of new biomarkers, based on the biology of the disease, is of major importance to HNSCC metastasis.

MicroRNAs, a small non-coding RNAs consisting of 20–22 nucleotides in length, have emerged as a new hotspot for anti-cancer therapy [6, 7]. By base-pairing complimentary or uncomplimentary sequences of 3′UTR of target mRNAs, microRNAs regulate gene expression in the post-transcription stages via mRNAs degradation or transcription inhibition. It has been suggested that miRNAs are capable to modulate a number of oncogenes or tumor suppressor genes and participate in the regulation of malignant transformation, progression of disease, and metastatic colonization [8]. Recently, a group of microRNAs have been identified altered in HNSCC and served as biomarkers for tumorigenesis, clinical prognosis, and metastasis [9–11].

MiR-34a, the most prevalent form of microRNA-34 family, is located in the region of chromosome lp36.23 and acts as a tumor-suppressor in variety of tumors [12]. It has been shown to represent direct target of p53 and repress several oncogenes directly or indirectly, resulting in cell-cycle arrest, senescence, apoptosis and reducing chemoresistance [13–15], and a miR-34 mimic has become the first microRNA to reach phase 1 clinical trials [12]. Recently, miR-34a is also reported as a tumor metastasis inhibitor in some solid cancers, but the target genes involved in metastasis have not been fully elucidated, and those targets vary in different cancer types [16–23]. To our knowledge, only one report has observed that miR-34a decreased HNSCC cell mobility, which might indicate its suppressing function in HNSCC metastasis [24]. However the role of miR-34a in metastasis of HNSCC has not been clearly elaborated, and until now, no target gene of miR-34a is determined in HNSCC. Therefore, exploring the function role and the possible target genes of miR-34a in HNSCC is an essential way to understand the molecular mechanism of miR-34a in tumor metastasis.

Human amphiregulin (AREG), synthesized as a 252-amino acid transmembrane precursor, pro-Areg, is an important ligand of the epidermal growth factor receptor [25]. A large number of information was available to support a role for AREG in tumor development and cancer cell biology. Studies have been demonstrated that over-expression of AREG participated in the maintenance of the oncogenic and metastatic properties in several solid malignancies through activation of EGFR families [26–29]. However, despite numerous identified stimuli, the epigenetic alterations responsible for the post-transcriptional regulation of AREG are not fully clarified [30], and information on the regulation of AREG expression by miRNAs is lacking [31].

In this study, we have identified that miR-34a acted as an essential metastatic suppressor in HNSCC with AREG as a novel target. Suppression of AREG by ectopic miR-34a expression prevented tumor invasion, and inhibited EGFR, which might be the potential molecular targets for future HNSCC therapy.

## RESULTS

### The expression of miR-34a in HNSCC samples

**Figure 1:**
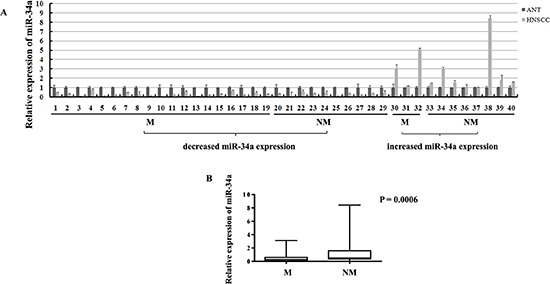
The expressions of miR-34a in HNSCC samples **(A)** The relative expressions of miR-34a in NHSCC tumors and the adjacent normal epithelial tissues. (M, lymph node metastatic samples; NM, non-metastatic samples). **(B)** The relative expressions of miR-34a in metastatic samples were significantly lower than that in non-metastatic samples.

In order to evaluate the expression of miR-34a in HNSCC tissues, real-time RT-PCR was performed in the human fresh specimens obtained from 40 primary HNSCC tumors and the adjacent normal epithelial tissues. About 42.0-fold to 1.3-fold reduction of miR-34a expression was found in 29 of 40 (72.5%) HNSCC tissues, compared with the adjacent normal epithelia (Figure 1A).

To determine the association between miR-34a expression and HNSCC metastatic potential, we investigated miR-34a expression in lymphatic metastasis samples and non-metastatic samples. Statistically, in the 29 cases of miR-34a reduction samples, 19 of which (65.5%) were associated with lymph node metastasis; 10 of which (34.5%) were associated with non-metastasis. In contrast, in the 11 cases of miR-34a increase samples, only 3 of which (27.3%) were associated with lymph node metastasis; 8 of which (72.7%) were associated with non-metastasis (Figure 1A). To normalize the relative expression value of miR-34a in cancer tissues, the miR-34a expression level in the corresponding matched adjacent normal epithelial tissues was defined as 1. Statistical analysis showed that the relative expression of miR-34a in metastatic samples (0.5685 ± 0.1847, mean ± SEM) was significantly lower than that in non-metastatic samples (1.274 ± 0.4602 mean ± SEM) (Figure 1B, *P* = 0.0006).

### MiR-34a suppresses migration and invasion of HNSCC cell lines *in vitro*

**Figure 2:**
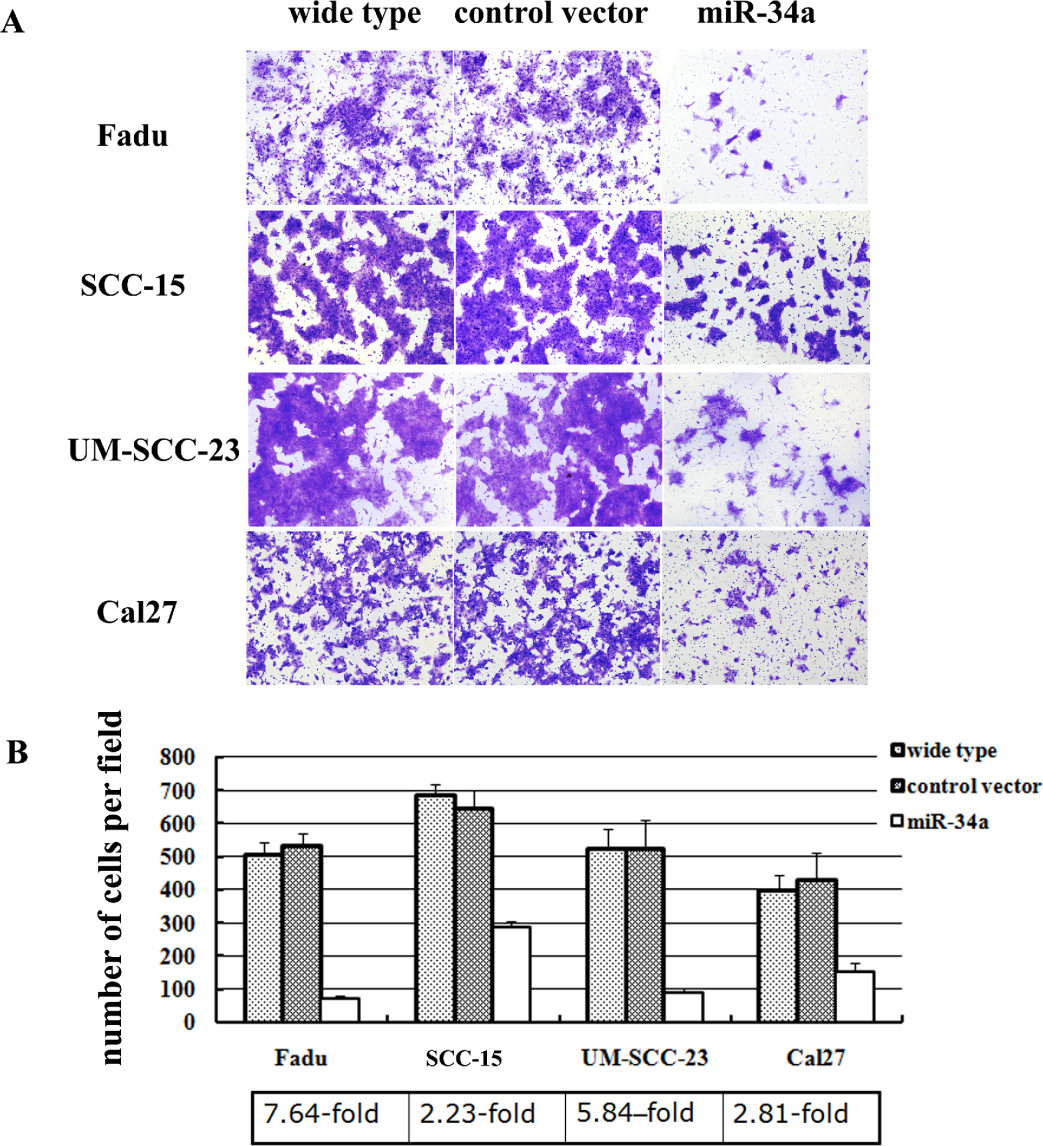
MiR-34a suppressed the invasion of HNSCC cell lines *in vitro* **(A)** The cell invasion capability was dramatically reduced in the cells over-expressed miR-34a. **(B)** Ectopic miR-34a resulted fold decrease in HNSCC cells invasion (*P* < 0.01).

To further elucidate the function of miR-34a in the metastatic process of HNSCC, four HNSCC cell lines (Fadu, SCC-15, UM-SCC-23, and Cal27) were stably over-expressed miR-34a using lentiviral vector. The miR-34a expression was enhanced dramatically in miR-34a transfected cells compared with the control (*p* < 0.01, Supplementary Figure 1). The ectopic miR-34a expression caused a significant reduction of cell invasion capability (Figure 2A). Statistically, over-expression of miR-34a resulted in 7.64-fold decrease in Fadu cell invasion, 2.23-fold decrease in SCC-15 invasion, 5.84-fold decrease in UM-SCC-23 invasion, and 2.81-fold decrease in Cal27, compared with the cells transfected with control vector (*P* < 0.01, Figure 2B).

### MiR-34a reduces metastatic potential of HNSCC cells *in vivo*

**Figure 3:**
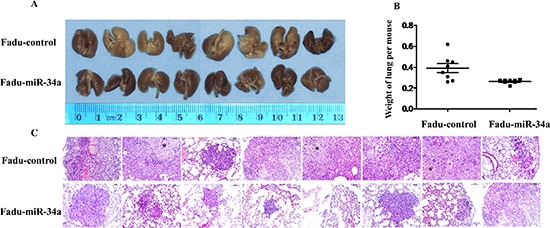
MiR-34a reduced metastatic potential of HNSCC cells *in vivo* **(A)** The macroscopy of metastasis nodes induced by Fadu-miR-34a and Fadu-control in the lung of nude mice. **(B)** The weights of mice lungs with metastasis nodes induced by Fadu-miR-34a and control cells (*p* < 0.01). **(C)** The histopathology of metastases induced by Fadu-miR-34a and Fadu-control in lung tissues with HE staining (Original magnifications × 100). *necrotic area.

To testify whether ectopic miR-34a could inhibit cell metastasis *in vivo*, Fadu cells over expressing miR-34a or transfected with control vector were injected into the tail vein of nude mice. Two months after tail vein injection, a dramatic effect on the size of eventually formed lesions and the weight of lung with metastases were observed. Fadu-miR-34a generated fine and scattered metastatic nodes, while the control cells (Fadu-miR-control) resulted in massive and confluent metastatic nodes (Figure 3A). Statistical analysis showed that the weights of mice lungs with metastatic nodes from Fadu-miR-34a cells shrunk about 1.5-fold compared with that from control cells (Figure 3B). The pathological changes of the bilateral lungs with HE staining were observed through light microscope at the same time. The tumor nests derived from control cells exhibited large area of lung tissue destruction and/or necrosis, while cells over-expressing miR-34a formed smaller and fewer tumor nests (Figure 3C).

### Identification of miR-34a targets by microarray analysis

**Figure 4:**
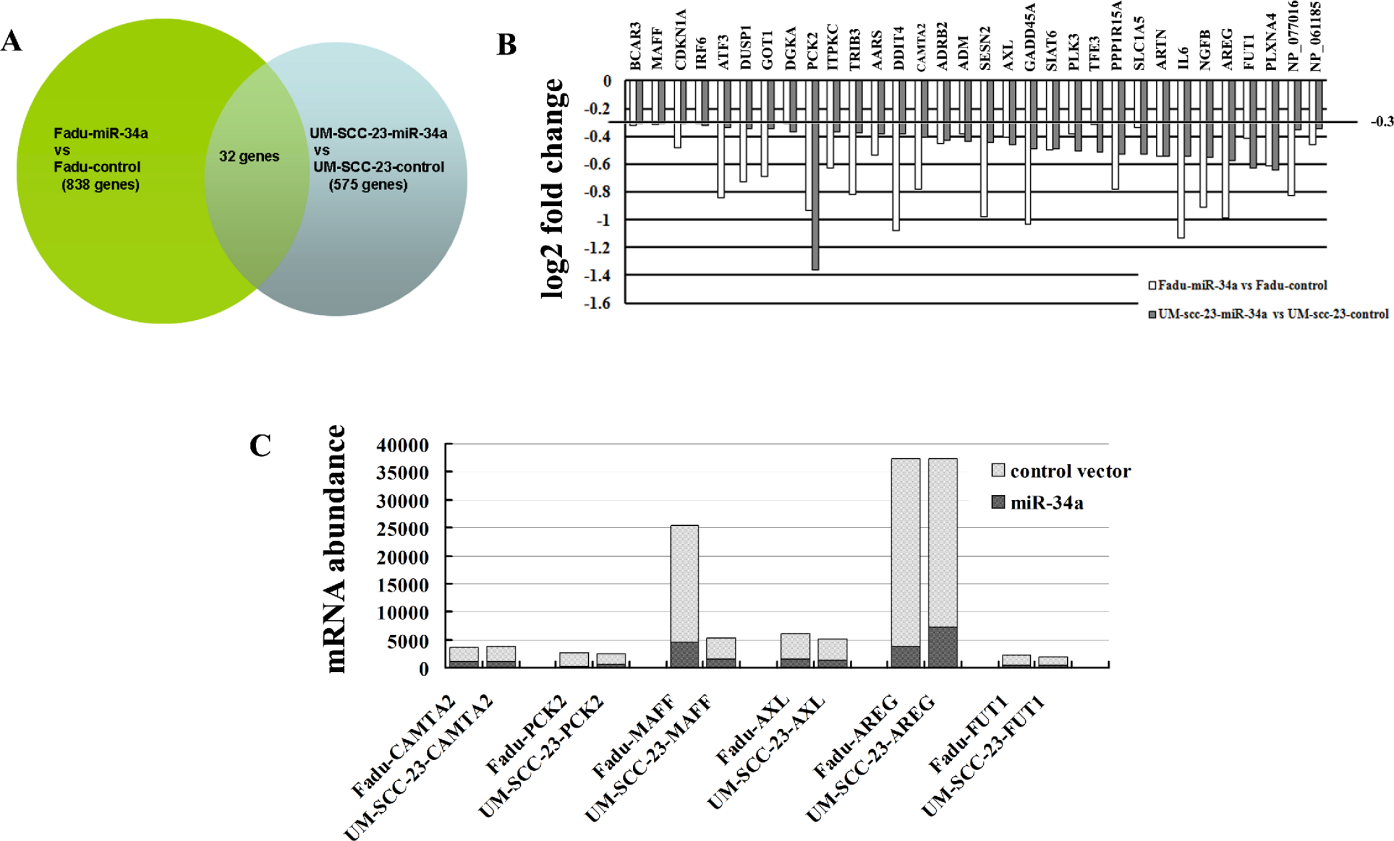
Identification of genes transcriptionally regulated by miR-34a through microarray analysis **(A)** Venn diagram of miR-34a mediated changes in genome wide mRNA expression. The overlap field showed the co-down-regulated genes. **(B)** Histogram showing co-down-regulated genes in mRNA levels after stable transfection ectopic pri-miR-34a in Fadu and UM-SCC-23 cells. Genes with a fold change log2 ≤ –0.3 were considered. **(C)** mRNA abundance of the candidate targets of miR-34a was detected by microarray analysis.

To study mechanisms responsible for suppression of HNSCC metastasis caused by ectopic miR-34a, we performed microarray and bioinformatic analyses to search for miR-34a target mRNAs. Genome wide mRNA expression was determined by two-channel microarray analysis in the following cell lines-Fadu-miR-34a (cy5)/control (cy3), and UM-SCC-23-miR-34a (cy5)/control (cy3). Thereby, transcriptional down-regulation of 838 genes and 575 genes were detected respectively in Fadu-miR-34a and UM-SCC-23-miR-34a cells compared with the control, when the differential mRNAs with a fold change log2 ≤ –0.3 were considered (Figure 4A). Combinedly, miR-34a over-expression resulted in 32 genes co-down-regulated in Fadu and UM-SCC-23 (Figure 4B). Among the co-down-regulated genes, 6 candidate mRNAs (CAMTA2, PCK2, MAFF, AXL, AREG, and FUT1) with seed-matching sequences in their 3′UTRs were predicted as miR-34a targets by using TargetScan, Pict-Tar and TargetScan5.2. According to TargetScan5.2, AREG and AXL have the conserved target site of miR-34a, while MAFF, FUT1, CAMTA2 and PCK2 show the poorly conserved target sites (Table 1). Through further analyzing the microarray data of 6 candidate genes, it was found that AREG showed not only the highest mRNA abundance in control cells (Fadu-control cy3 intensity = 33543, UM-SCC-23 cy3 intensity = 30178), but also the largest log2 fold decrease in miR-34a over-expressed cells (Fadu-miR-34a/Fadu-control: cy5/cy3 intensity log2 change = –0.99, UM-SCC-23-miR-34a/UM-SCC-23-control: cy5/cy3 intensity log2 change = –0.57) (Figure 4C).

**Table 1:** The candidate target genes of miR-34a predicted by TargetScan5.2

Genes	Predicted consequential pairing of target region (top) and miR-34a (bottom)	Conserved site	Poorly conserved site
AREG 3′UTR(35–42)	5′…AUAUCACAUUGGAGUCACUGCCA…3′ UGUUGGUCGAUUCUGUGACGGU	1(7mer-1A)	
AXL 3′UTR(44–51)	5′…AGGAUCCAAGCUAAG - CACUGCCA… 3′ UGUUGGUCGAUUCUGUGACGGU	1(8mer)	
MAFF 3′UTR(1027–1033)	5′…CCCCAACACUGUCCACACUGCCC… 3′ UGUUGGUCGAUUCUGUGACGGU		1(7mer-m8)
PCK2 3′UTR(2–8)	5′…NNNNNNNNNNNNNNNGACUGCCAG… 3′ UGUUGGUCGAUUCUGUGACGGU		1(7mer-1A)
CAMTA2 3′UTR(377–383)	5′…CGCCUCGGUUCCCACCACUGCCU… 3′ UGUUGGUCGAUUCUGUGACGGU		1(7mer-m8)
FUT1 3′UTR (1567–1574)	5′…CGUGGGGUGAGGGAUCACUGCCA… 3′ UGUUGGUCGAUUCUGUGACGGU		2(8mer, 7mer-m8)

### AREG is a direct target of miR-34a

**Figure 5:**
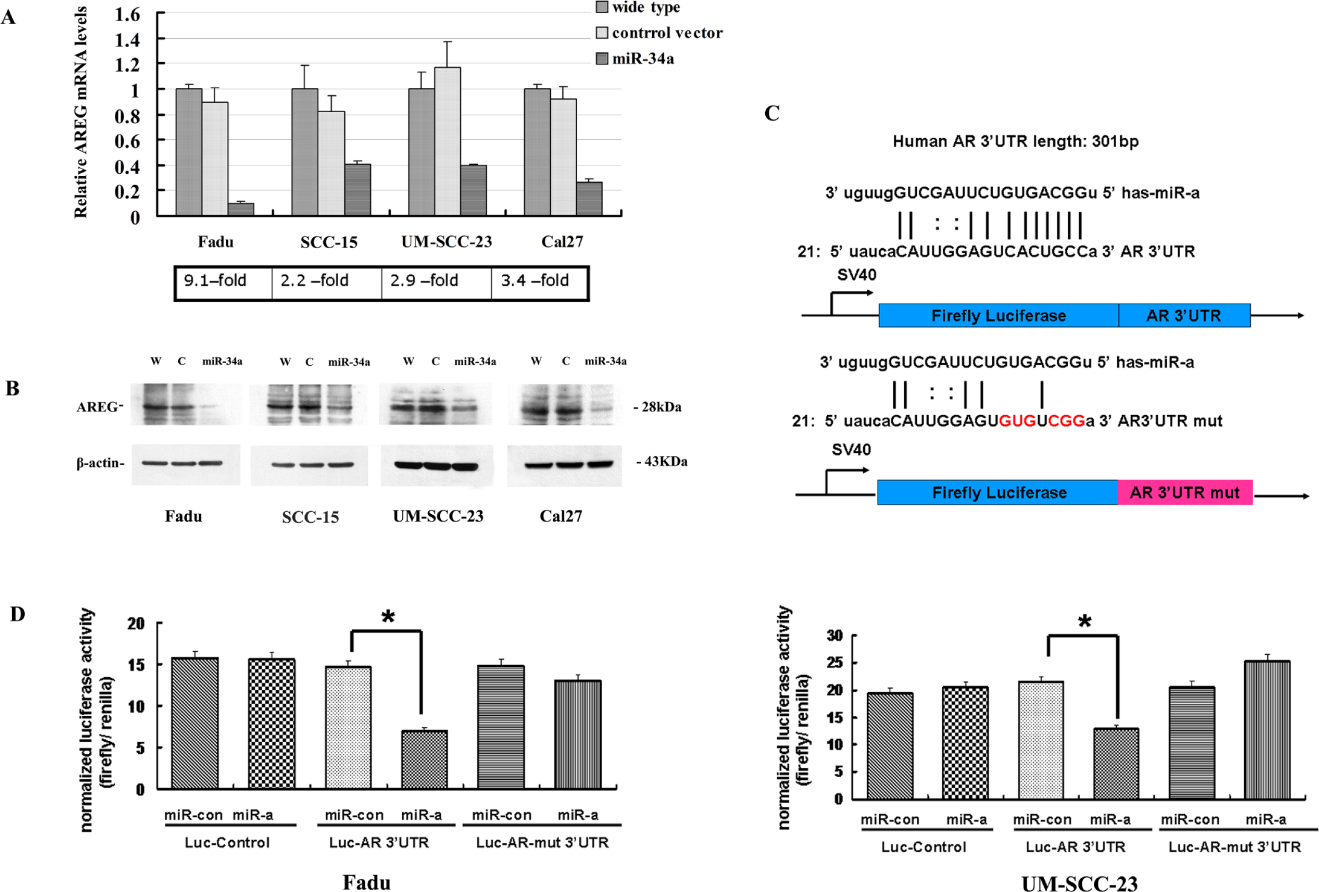
AREG was identified as a direct target of miR-34a **(A)** AREG-transcript levels had a generally reduction in HNSCC cell lines with ectopic miR-34a expression. **(B)** The reduction of AREG protein amounts in miR-34a over-expression cell lines. **(C)** The 3′-UTR sequence of AREG and the mutant AREG 3′-UTR sequence were cloned into pSiCheck™-2 firefly luciferase vector. **(D)** Forced expression of miR-34a reduced the luciferase reporter activity of pSiCheck™-2-AREG 3′-UTR (*p* < 0.01), while had a minimal effect on pSiCheck™-2-AREG-mutant 3′-UTR.

To identify whether the expression of AREG is suppressed by miR-34a, the mRNA expression level of AREG gene was screened by qRT-PCR in four HNSCC cell lines. The results showed that AREG-transcript levels had a general reduction in HNSCC cell lines with ectopic miR-34a expression compared with control (9.1-fold decrease in Fadu-miR-34a, 2.2-fold decrease in SCC-15-miR-34a, 4.9-fold decrease in UM-SCC-23-miR-34a, and 3.4-fold decrease in Cal27-miR-34a) (Figure 5A). Furthermore, western blot analysis confirmed the ectopic miR-34a expression causing a remarkable reduction of AREG protein amounts in those cell lines (Figure 5B).

To further validate whether AREG is the direct target of miR-34a, we performed dual reporter assay using reporter constructs containing 3′-UTR sequences of AREG, and mutated the miR-34a seed-marching sequence in AREG 3′-UTR (Figure 5C). Forced expression of miR-34a reduced the luciferase reporter activity of pSiCheck™-2-AREG 3′-UTR by about 40–50%, compared with the miR-control (*p* < 0.01, Figure 5D). In contrast, miR-34a had a minimal effect on the reporter activities of the pSiCheck™-2-AREG-mutant 3′-UTR (Figure 5D).

### Inhibition of AREG expression impaired HNSCC cell invasion *in vitro*

**Figure 6:**
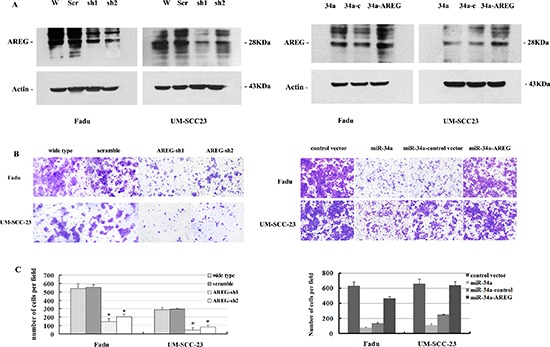
(A) Attenuated AREG protein expression by AREG mRNA knocking down **(B, C)** Inhibition of AREG expression impaired HNSCC cell invasion *in vitro*. **(D)** Increased AREG protein expression by over-expression of AREG mRNA level. **(E, F)** Re-expression of AREG partially rescues miR-34a-imposed cell invasion defects *in vitro*.

To assess the functional contributions of AREG to HNSCC invasion, we first examined whether AREG inhibition affected the invasion of Fadu and UM-SCC-23 cells. Stable clones of Fadu-AREG-sh1/-sh2 and UM-SCC-23-AREG-sh1/sh2 were generated that expressed AREG shRNA (AREG-sh1/-sh2) and exhibited diminished AREG protein expression (Figure 6A). Attenuated AREG expression in Fadu-AREG-sh and UM-SCC-23-AREG-sh cells caused a significant decrease in HNSCC cell invasion (Figure 6B). Statistically, repressing AREG decreased Fadu cells invasion by 2.7~3.7-fold and UM-SCC-23 cells invasion by 3.4–6.6-fold (*p* < 0.01, Figure 6C).

### Re-expression of AREG partially rescues miR-34a-imposed cell invasion defects *in vitro*

To determine whether restoration of AREG level could reverse miR-34a overexpression-dependent tumor invasion defect, we stably over-expressed AREG gene inFadu-miR-34a and UM-SCC-23-miR-34a cells using pBABE retrovirus expression vector encoding AREG CDS sequence (pBABE-AREG). Then, stable clones of Fadu-miR-34a-AREG and UM-SCC-23-miR-34a-AREG were generated and exhibited a significant increase in AREG mRNA and protein level (Figure 6D, Supplementary Figure 2). Forced AREG expression promoted about 3.3-fold increase of *in vitro* invasion in Fadu-miR-34a cells, and about 2.6-fold increase of invasion in UM-SCC-23-miR-34a cells (*p* < 0.01, Figure 6E and 6F). The result demonstrated that re-expression of AREG partially rescued miR-34a-mediated invasion defects.

### Re-expression of AREG reverses miR-34a-imposed metastasis defects *in vivo*

**Figure 7:**
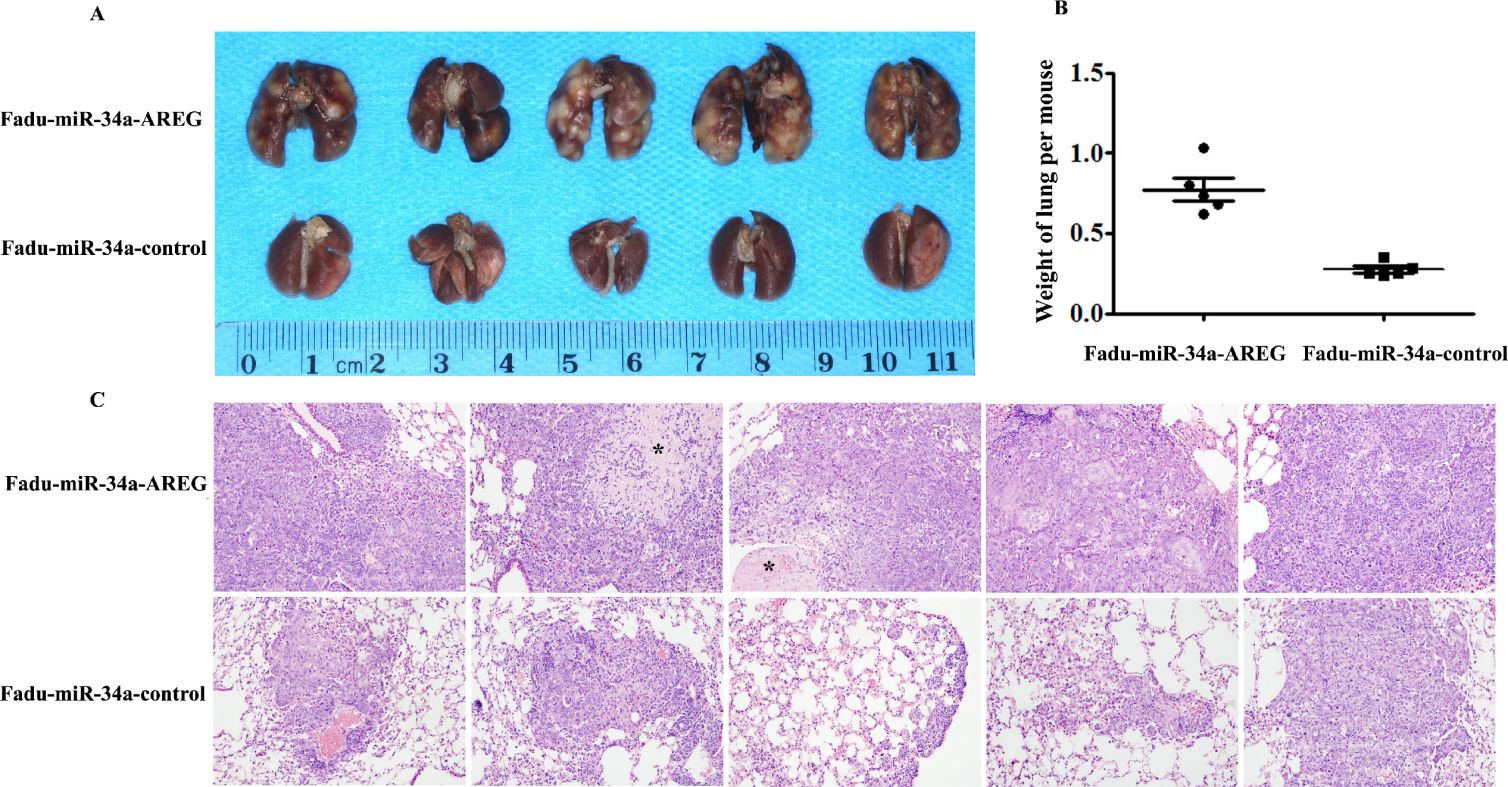
Re-expression of AREG reverses miR-34a-imposed metastasis defects *in vivo* **(A)** The macroscopy of metastasis nodes induced by Fadu-miR-34a-AREG and Fadu-miR-34a-control in the lung of nude mice. **(B)** The weights of mice lungs with metastasis nodes induced by Fadu-miR-34a-AREG and control cells (*p* < 0.01). **(C)** The histopathology of metastases induced by Fadu-miR-34a-AREG and control in lung tissues with HE staining (Original magnifications × 100). *necrotic area.

To ascertain whether restored AREG reversed *in vivo* tumor metastasis defects caused by miR-34a, stable clones of Fadu-miR-34a-AREG and Fadu-miR-34a-control cells were injected into the tail vein of nude mice, respectively. Two months later, the macro and microscopic changes of the mice bilateral lungs were evaluated. In consonance with our earlier findings, ectopic miR-34a generated fine and scattered metastatic nodes; in contrast, Fadu-miR-34a-AREG cells generated masses of metastatic nodes in mice lungs (Figure 7A and 7C). Statistical analysis showed that the weights of mice lungs with metastatic nodes from Fadu-miR-34a-AREG cells were about 2.8-fold higher than those from control cells (Figure 7B).

### EGFR are inhibited by ectopic miR-34a through suppressing AREG

**Figure 8:**
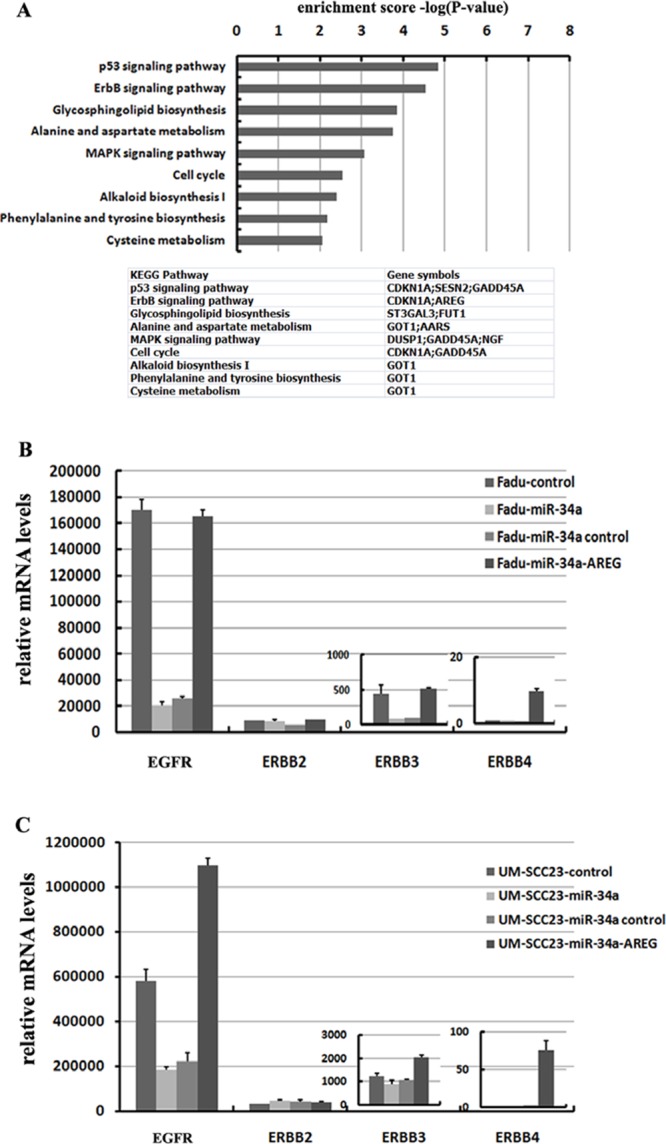
miR-34a involves in ErbB pathway though suppressing AREG **(A)** 32 co-down-regulated mRNAs with log2 fold change ≤ –0.3, were subjected to KEGG pathway enrichment analysis. **(B)** Re-expression AREG reversed miR-34a induced EGFR and ErbB3 down-regulation in Fadu cell lines. **(C)** Re-expression AREG reversed miR-34a induced EGFR down-regulation in UM-SCC-23 cell lines.

The 32 co-down-regulated genes affected by forced miR-34a expression were performed by a KEGG pathway analysis. The result showed that the differentially regulated genes involved in P53 signaling pathway (*p* = 1.43E-05) and ErbB signaling pathway (*p* = 2.86E-05) were over-represented among the down-regulated mRNAs (log2 fold change ≤ –0.3, *p* < 0.01). Besides the well-known P53 signaling pathway, the influence of miR-34a in ErbB pathway is also important (Figure 8A).

To identify which of the ErbB family members were inactivated by ectopic miR-34a expression through inhibiting AREG, four EGF receptors (EGFR, ErbB2, ErbB3 and ErbB4) were detected. In HNSCC cell lines, the expression level of EGFR (ErbB1) was extremely high, in contrast, ErbB4 were fairly low. Forced miR-34a expression significantly decreased EGFR and ErbB3 mRNA levels in Fadu cell line, while only EGFR was remarkably suppressed in UM-SCC-23 cell line (Figure 8B and 8C). The changes of ErbB2 and ErbB4 mRNA levels were very slight in response to miR-34a over-expression. Interestingly, re-expression AREG in miR-34a over-expression cell lines not only dramatically elevated EGFR, but also ErbB3 and ErbB4 mRNA levels (Figure 8B and 8C).

### uPA is inhibited by miR-34a through suppressing AREG

**Figure 9:**
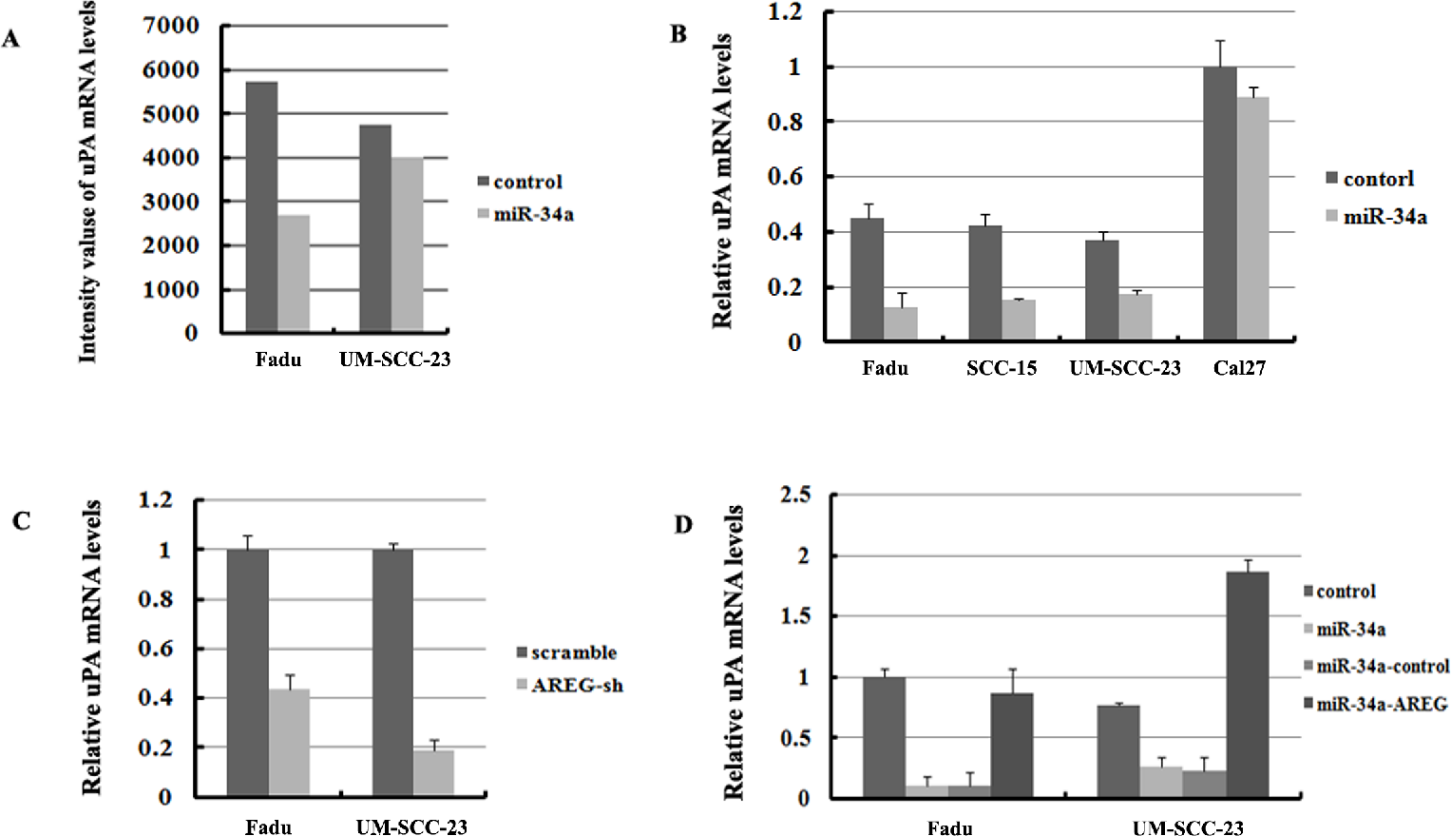
UPA is regulated by miR-34a though inhibition of AREG **(A)** mRNA abundance of uPA detected by microarray analysis. **(B)** The relative expression of uPA mRNA were detected by qRT-PCR in four HNSCC cell lines. **(C)** Inhibition of AREG expression significantly suppressed uPA mRNA level. **(D)** Forced AREG expression rescued the uPA mRNA level.

Based on the microarray results, uPA (also called PLAU) the downstream factor of ErbB pathway was found decreased in Fadu-miR-34a (log2 change = –0.311) and UM-SCC-23-miR-34a (log2 change = –0.104) (Figure 9A). The decrease of uPA was verified by qRT-PCR screend in four HNSCC cell lines, and three of four cell lines showed that miR-34a over-expression significantly reduced uPA mRNA levels (*p* < 0.01, Figure 9B). Although the log2 change of uPA in UM-SCC-23-miR-34a was higher than –0.3 according to microarray data, qRT-PCR result demonstrated uPA decreased about 2.14-fold in UM-SCC-23-miR-34a, compared with the control. Moreover, inhibition of AREG expression also significantly suppressed uPA mRNA levels (*p* < 0.01, Figure 9C). On the other hand, forced AREG expression in Fadu-miR-34a and UM-SCC-23-miR-34a cells effectively rescued the expression of uPA mRNA (*p* < 0.01, Figure 9D). The results might suggest that ectopic miR-34a suppresses uPA by inhibiting AREG.

### Expression of AREG is partially inversely correlated with miR-34a in HNSCC samples and associated with metastatic phenotypes

**Figure 10:**
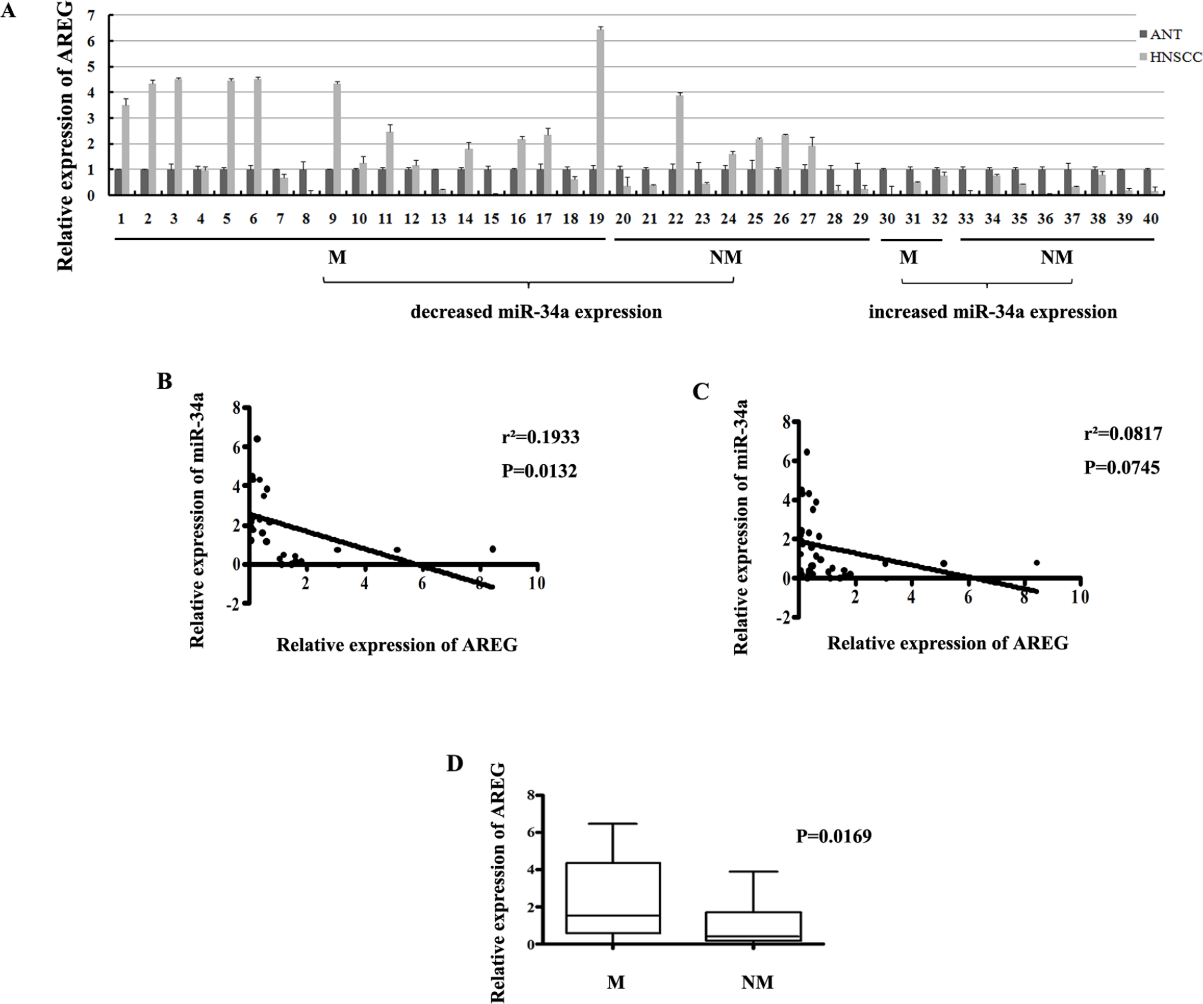
(A) The relative expression of AREG mRNA in NHSCC tumors and the adjacent normal epithelial tissues **(B)** The relative expression of AREG mRNA in metastatic samples was significantly higher than that in non-metastatic samples. **(C)** General correlation between miR-34a and AREG mRNA relative expression in 40 samples. **(D)** The inverse correlation between miR-34a and AREG mRNA relative expression in 29 samples.

To investigate whether AREG expression was inversely correlated with miR-34a in HNSCC tissues, the mRNA expression of AREG was evaluated by real-time RT-PCR in the 40 primary HNSCC tumors and the corresponding adjacent normal epithelial tissues. There were 29 of 40 (72.5%) HNSCC samples showed opposite expression trend between AREG and miR-34a (Figure 10A). Statistically, the relative AREG expression was negatively correlated with miR-34a in the 29 samples (Figure 10B, *P* = 0.0132), although general inverse correlation between miR-34a and AREG expression in all 40 tumor tissues was not observed (Figure 10C, *P* = 0.0745).

To determine the association between AREG expression and HNSCC metastatic potential, we investigated AREG mRNA level in lymphatic metastasis samples and non-metastatic samples. The relative expression level of AREG in metastatic samples (2.147 ± 0.4063, mean ± SEM) was much higher than that in non-metastatic samples (0.9086 ± 0.2493, mean ± SEM) (Figure 10D, *P* = 0.0169). Furthermore, the reversed expression pattern between miR-34a and AREG was found to be closely related with HNSCC lymph node metastasis. It was shown that miR-34a level was decreased and AREG mRNA level was simultaneously increased in 18 samples, 13 of which (72.2%) showed lymph node metastasis. On the other hand, 11 samples co-expressed increased miR-34a and decreased AREG, 3 of which (27.3%) exhibited lymph node metastasis (Figure 10A). The results indicate that patient who loses miR-34a expression meanwhile over-expresses AREG, tends to exhibit highly potential of lymphatic metastasis, and vice versa.

## DISCUSSION

Head and neck squamous cancer is a heterogeneous oncological setting typically appears in the oral cavity, oropharynx, hypopharynx, or larynx. Local invasion and lymph node metastases are the major causes of recurrence and mortality of HNSCC [32]. For those patients in recurrent/distant metastases stages, multimodality therapy approaches with surgery, radiotherapy, and chemotherapy, have reached their upper limit of tolerability. The mortality rate of patients presented with advanced disease is higher than expected [33]. To date, although cetuximab has been approved for commercial use for molecular-targeted therapy in HNSCC, only a minority of patients with stage III/IV HNSCC have meaningful tumor regression [4, 5]. Hence, the investigation of new molecular targets, based on the biology of HNSCC, will be important to improve treatment efficacy. Here, we demonstrated that miR-34a through targeting amphiregulin, a ligand of EGFR, was a distinct signaling pathway in inhibition of HNSCC metastasis, which might be a potential therapeutic target of HNSCC.

MiR-34a is best understood as a pleiotropic anti-cancer microRNA, which acts majorly on cell-cycle arrest, differentiation, senescence and apoptosis [12, 34]. In the last few years, studies further discovered that ectopic overexpression of miR-34a results in the inhibition of metastasis in some solid tumors, including colon cancer [16, 35], prostate cancer [17], hepatoma [19], non-small cell lung cancer [18], endometrial carcinoma [36] and osteocarcinoma [37]. In head and neck cancer, one previous study observed that overexpression of miR-34a may decrease the cell migration motility *in vitro* [24]. This result may suggest a potential effect of miR-34a on HNSCC invasion. However, more detailed evidences should be given to fully elaborate whether the miR-34a has an effective function in blocking HNSCC metastasis. Our present study demonstrated that the miR-34a expression was reduced in HNSCC when compared with the adjacent normal epithelial tissues. Moreover, the downregulation of miR-34a was mainly found in tumors with lymph node metastasis, suggesting an association between the loss of miR-34a and the potential of HNSCC lymph node metastasis. Moreover, forced expression of miR-34a dramatically decreased HNSCC cells invasion *in vitro*, and strikingly impaired their capacity to seed lung metastases *in vivo*. These results further confirmed the effective function of miR-34a in suppressing HNSCC invasion and metastasis.

Identification of miRNA target gene is of important approach to understand the molecular mechanisms of miRNA-mediated tumorigenesis. Up to now, miR-34a is reported to target over fifty genes directly, most of which are involved in inhibition of cell cycle and apoptosis [38–40]. There are several targets of miR-34a related to metastasis have been identified, including Fra-1 [16], AXL [18], CD44 [17], c-Met [19], SNAIL [41], ARHGAP1 [21], AXIN2 [22], c-Myc [23] and YY1 [42]. It is noted that miR-34a usually targets different genes in different tumor types, suggesting miR-34a may involve in distinct signaling pathway in specific tumor. In our study, in order to screen the possible target genes of miR-34a in HNSCC, a microarray-based differential mRNA profiling using cells transfected with miR-34a or control vector was performed. Then, AREG was selected and finally confirmed to be a crucial target of miR-34a through a series of verified experiments. We provide evidence for decreased expression of the AREG gene at the mRNA and protein levels when forced expressing of miR-34a in four HNSCC cell lines. In particular, miR-34a reduced the luciferase reporter activity of the pSiCheck™-2-AREG-3′-UTR, while had a minimal effect on the reporter activities of the pSiCheck™-2-AREG-mutant 3′-UTR, confirming that miR-34a degrades AREG gene through binding on the seed sequence in AREG 3′-UTR. Recently M.A. Avila has predicted microRNAs might involved in regulation of AREG mRNA, however the clear information on the miRNAs in AREG mRNA regulation has not been ever reported [31]. To our knowledge, this is the first time that miR-34a is demonstrated to inhibit tumor metastasis in HNSCC by directly targeting AREG, which provides the evidence that AREG could be post-transcriptionally regulated by microRNA. On the other hand, based on our microarray data, there are several other candidate genes have potential miR-34a binding site on the 3′-UTR, such as AXL, MAFF, FUT1, CAMTA2 and PCK2. Among these, AXL and FUT1 were previously determined as the targets of miR-34a in non-small cell lung cancer, colorectal cancer and breast cancer [18, 43]. Unfortunately, none of them have a universal decrease like AREG in four HNSCC cell lines over-expressing miR-34a (data not shown). Since microRNA could post-transcriptionally regulate gene expression by mRNA degradation or translation inhibition or both, loss of miR-34a may activate more than one pathway involved in HNSCC metastasis. Future efforts on identifying more targets of miR-34a should be developed by combining more advance approaches to clarify the mechanisms of miR-34a in HNSCC progression, like using high-throughput mRNA and Proteomics sequencing combined with computational algorithms or bioinformatics.

AREG, short for amphiregulin, synthesized as a 252-amino acid transmembrance precursor pro-Areg, is one of a member of the epidermal growth factor family [44]. The family pairs with and induces the homo- or heterodimerization of four receptors: ErbB1 (EGFR), ErbB2, ErbB3 and ErbB4 [45–47]. The extracellular domain is the least conserved among the 4 ErbB members, allowing AREG specifically bind to the EGFR resulting in phosphorylation of C-terminal tail, and subsequent downstream signaling [30]. Upon binding to EGFR, AREG can also induce the activation of ErbB2, ErbB and ErbB4 [31]. In accordance with previous researches, our data showed that EGFR was significantly inhibited by abundant miR-34a or defective AREG, besides which ErbB3 and ErbB4 were also remarkably activated by restoration of AREG in miR-34a over-expressed cells. It suggested that miR-34a may play an important role in regulation of ErbB pathway though inactivating ErbBs via targeting AREG. In addition, AREG is found to be less effective in inducing ErbB receptors at low concentrations, but has an affinity for the receptors at higher concentrations [30]. These might partially explain why re-expression AREG in miR-34a over-expression cell lines not only dramatically elevated EGFR, but also ErbB3 and ErbB4 mRNA levels.

Recent decade, studies have demonstrated AREG can indeed behave as a pro-oncogenic factor and highlighted the multiple functional roles of AREG over-expression in inhibition of apoptosis and promoting proliferation, motility, angiogenesis, and tumor invasion and metastasis in many solid tumors [31, 48, 49]. Besides the direct effects that autocrine or paracrine, a recent study has identified exosome-mediated AREG signaling in human breast and colorectal cancer cells, which activates EGFR resulting cell invasion [50]. Most importantly, AREG was also identified to participate in the tumor resistance to PI3K inhibition and sorafenib therapy, and as the sole targeted agent in the treatment of advanced solid carcinomas [51, 52]. In our research, the cellular effects of AREG in metastasis were identified in HNSCC that AREG over-expression was strongly associated with HNSCC metastatic phenotype in the tissues and cell lines. Attenuated AREG expression significantly decreased HNSCC invasion *in vitro*, and restoration of AREG rescued miR-34a-imposed cell invasion and metastasis defects *in vitro* and *in vivo*. The results agree with the previous study that knockdown of pro-AREG expression by SiRNAs inhibited the migration of SCC-9 head and neck cancer cells toward fibronectin [53]. Moreover, for the first time, AREG was identified to promote *in vivo* tumor metastasis and reverse miR-34a-imposed metastasis defects, indicating that AREG is the key determinant in the maintenance of metastatic properties of HNSCC, and a potential therapeutic target for metastatic tumor.

The urokinase plasminogen activator (uPA) is one of the serine proteinase systems involved in ECM degradation, which are overexpressed in several malignant tumors. For decades, the urokinase plasminogen activator (uPA) system has been thought to drive tumor progression by mediating directed extracellular proteolysis on the surface of migrating or invading cells. Intervention with this proteolysis by targeting uPA system has been proposed to represent a novel approach for inhibiting tumor progression and a potential value as molecular prognostic marker prostate cancer, melanoma, breast cancer, pancreatic cancer, gastric cancer, thyroid cancer, and etc [54–58]. Previous researches also showed that the downstream factors of ErbB pathway, such as MMP-9, uPA, EMMPRIN, and PAI-1, could be induced by AREG in head and neck cancer [28, 29], breast cancer [59, 60], and malignant mesothelioma cell lines [61]. Although both of MMP-9 [28, 62] and uPA [29] were demonstrated previously activated by AREG in HNSCC invasion and metastasis, based on our microarray data and qRT-PCR results, only uPA showed significant decreases in this research, when AREG was suppressed by ectopic miR-34a. Our results indicate that degradation of uPA by inhibition of AREG might be an essential pathway of miR-34 modulating HNSCC invasion defects.

In conclusion, our data show that miR-34a has a pivotal role in suppressing HNSCC invasion and metastasis. AREG is one of essential miR-34a targets, over-expression of which could rescue miR-34a mediated tumor invasion defects and EGFR inhibition. Patients, who lose miR-34a expression and over-express AREG, tend to exhibit highly potential of lymphatic metastasis. Therefore, miR-34a/AREG might be promising molecular targets in anti-invasion/metastasis of HNSCC.

## METHODS

### Cell culture and tumor samples

The head and neck cell line, UM-SCC-23, was obtained from University of Michigan (a gift of Dr. Thomas E. Carey, the cell line MAT from University of Michigan in 2012.06). The Cal27 derived from a tongue SCC patient was a gift of Dr. Xinhong Wang (Medical college of Guangzhou). The Fadu cell line was derived from a hypopharynx SCC patient (a gift of Xinhong Wang, Medical college of Guangzhou). The SCC-15 derived from a tongue SCC patient was obtained from the American Type Culture Collection (ATCC, Manassas, VA, USA). The HEK-293T embryonic kidney cell line was obtained from the American Type Culture Collection (China Center for Type Culture Collection CCTCC, Wuhan, Hubei, China). Fadu, UM-SCC-23, Cal27 and HEK-293T cells were cultured respectively in DMEM medium (Invitrogen, Carlsad, CA). SCC-15 cells were cultured in DMEM:F12 (1:1) medium (Invitrogen, Carlsad, CA). The cell culture medium was supplemented with 10% fetal bovine serum (FBS) with 1000 units/mL penicillin and 0.1 mg/mL streptomycin. The cell lines from other labs were performed the DNA (STR) profiling to avoid being misidentified and cross-contaminated by Microread Genetics (Beijing Microread Genetics Co; Ltd. Beijing, China) (Supplementary Figure 3).

40 cases of fresh primary HNSCC samples and the corresponding matched normal epithelial tissues were obtained during surgery at the Stomatology Hospital of Wuhan University between 2012–2013 (including 22 tongue SCCs, 11 oropharynx SCCs, 4 buccal mucosa SCCs, 2 mouth floor and 1 gingival SCC). None of the patients received chemotherapy before surgery. Tissue samples were treated with RNA safe solution (Promoter biotechnology, Wuhan, China) immediately and stored at –80°C before use. The Chinese Ethic Committee approval was received for the examination of patient samples. Patients were HPV-16 negative based upon HPV16 detection by PCR. Total RNA was isolated from the HNSCC samples and corresponding matched normal epithelial tissues using the mirVana miRNA isolation kit (Ambion, Taxas, USA).

### Plasmids and stable transfection

For miR-34a overexpression study, the lentivirus expression vector for pri-miR-34a overexpression system (pCDH-EF1-copGFP-pre-miR-34a and pCDH-EF1-copGFP-control), was purchased from Systems Biosciences.

For AREG over-expression study, AREG gene was PCR-amplified according to AREG CDS sequence (NM_001657.2). The primer pair was as follows: 5′ATGAGAGCCCCGCTGCTACCGCC3′, and 5′TTATGCTATAGCATGTACATTTCCA3′. The pBABE-puro lentiviral vector was purchased from Addgene (Addgene, Cambridge, USA). PBABE-AREG plasmid was generated by inserting AREG CDs sequence into the BamH1 and EcoR1 sites of pBABE vector and verified by sequencing.

For gene knockdown study, the pLKO.1 and pLKO.1-scramble lentiviral vector were purchased from Addgene (Addgene, Cambridge, USA). The pLKO.1-AREG-sh1 and pLKO.1-AREG-sh2 plasmids were generated by inserting the oligonucleotide containing the specific shRNA target sequence into the pLKO.1 vector and verified by sequencing. The shRNA sequence pairs were as follows: AREG-sh1, 5′CCGG*CCACAGTGCTGATGGATTT*CTCGAG *AAATCCATCAGCACTGTGG*TTTTTG3′, and 5′AATTCAAAAA*CCACAGTGCTGATGGATTT*CTCGAG*AAATCCATCAGCACTGTGG*3′; AREG-sh25′CCGG*GCTGATGGATTTGAGGTTA*CTCGAG*TAACCTCAAATCCATCAGC*TTTTTG3′, and 5′AATTCAAAAA*GCTGATGGATTTGAGGTTA*CTCGAG*TAACCTCAAATCCATCAGC*3′.

For each plasmid to be transfected, plate HEK-293T cells were seeded in a 10 cm tissue culture plate and incubated at 37°C, 5% CO_2_ overnight. When HEK-293T cells get 50%–70% confluent in the next day, a cocktail for each transfection was made by putting the constructed plasmid, packaging plasmid, and envelope plasmid to serum-free OPTI-MEM. Drop wise the cocktail media to HEK-293T cells and incubate cells at 37°C, 5% CO2 for 24 hours and change the media in the next day. 2 days later, harvest the virus and store in the –80°C. To stably transfect the target cells, lentiviral particle solution with polybrene was added to the 80–90% confluent target cells. Change to fresh media 24 hours after infection and use GFP fluorescence sorting or puromycin to select transfected cells.

### Quantitative reverse-transcription PCR (qRT-PCR)

Total RNA was extracted from the HNSCC cell lines using the mirVana miRNA isolation kit (Ambion, Taxas, USA). TaqMan microRNA assay specific for miR-34a (Assay ID 000426, Applied Biosystems, Carlsband, CA) was used to detect and quantify mature miR-34a according to the manufacturer's protocol (Applied Biosystems, Carlsband, CA) using an Applied Biosystems 7500 Detection system. For AREG, EGFR, ErbB2, ErbB3, ErbB4 and uPA qRT-PCR, RNA was transcribed into cDNA using PrimeScript^®^ RT reagent Kit (Takara Code:DRR037A, Dalian, Japan), and the gene expression was quantified by SYBR^®^ Premix Ex Taq™ GC Kit (Takara Code: DRR091A, Dalian, Japan). The primer sets as follows were prevalidated to give single amplicons: AREG: 5′TGTCGCTCTT GATACTCGGC3′, and 5′AGGCATTTCACTCACA GGGG3′; EGFR: 5′TTGCCGCAAAGTGTGTA ACG3′, and 5′GAGATCGCCACTGATG GAGG3′; ErbB2: 5′CCAGAATGGCTCAG TGACCT3′, and 5′TCCAGATGGGCATGTAGG AGA3′; ErbB3: 5′CATCGTGAGGGACCGA GATG3′, and 5′TCATCATGGCAGCACTGGTT3′; ErbB4: 5′TGACTCGAATAGGAACCAG TTTGT3′, and 5′GCCACAGGAGCTTCTGGAAT3′; uPA: 5′ACTCCAAAGGCAGCAATGAAC3′, and 5′ATTTCACAGTGCTGCCCTCC3′. The cycling parameters were 95°C, 15 min; 40 cycles of (95°C, 15 s; 55°C, 30–40 s and 72°C, 30 s). miRNA and mRNA expression was normalized to RNU6B and *β*-actin, respectively. The different expression of miRNA and mRNA in Control-miR or miR-34a samples was deducted from 2-ΔΔCt where ΔΔCt = ΔCt miR-34a overexpression cells–ΔCt Control-miR cells.

### Cell invasion assay

BD Matrigel™ Basement Membrane Matrix (BD Biosciences Inc. San Jose, CA, USA) was thawed on ice at 4°C and diluted with the ratio of 1:8 with DMEM. 30 ul of the diluted BD Matrigel matrix coating solution was added to the 6.5 mm transwell with 8.0 um pore polycarbonate membrane insert (BD Biosciences Inc. San Jose, CA, USA) and incubated at 37°C for 5–6 hours. For the cell invasion assay, 1.5 × 10^5^ cells were seeded onto a Matrigel-coated chamber with 8.0 mm pores (BD Biosciences Inc. San Jose, CA, USA). Cancer cells were seeded using serum-free media to the upper chamber pairing with the lower chamber filled with 10% serum DMEM media. At 24 hrs after allowing cells to invade, cells on the lower side of the chamber were fixed and stained with crystal violet (Promoter biotechnology, Wuhan, China). The total number of cells per high-power microscopic field on the lower side of matrigel-coated chamber was counted and scored for migrating or invading cells. The mean number of cells in five high-power microscopic fields was calculated with standard deviations.

### *In vivo* metastatic assay

Fadu cells infected with lentiviruses carrying control vector or miR-34a expression construct, or miR-34a-AREG expression construct were used for *vivo* pulmonary metastasis assays. 6–8 weeks old female BALB\c-nu nude mice (animal experiment center, Wuhan University, China) were injected with 1 × 10^6^ cells (resuspended in 100 μL PBS) via the tail vein. Eight weeks after the injection, the mice were sacrificed and the lung tissues were isolated. Metastasis was quantified by evaluating the weights of lung tissues. The lung tissues were made into serial sections before HE staining. The pathological changes were observed under a light microscope. Mice were manipulated and housed in accordance with the Guide for the Care and Use of Laboratory Animals (Ministry of Science and Technology of China, 2006), and were approved by the animal ethics committee of Wuhan University. All efforts were made to minimize suffering.

### MRNA microarray analysis

Total RNA was extracted from Fadu and UM-SCC-23 cells expression of miR-34a and control miR-vector, and was further purified with an RNA clean-up kit (Macherey-Nagel, Germany). RNA was quantified by using of spectrophotometer and then was assessed by visualization of the 28S/18S ribosomal RNA ratio on 1.2% formamide agarose gel. Acceptable values were defined as: A260/A280 ratio in the range of 1.8–2.2, rRNA ratio (28S/18S) > 0.9 and RIN value > 8.0. cRNA (2 mg) was reversely transcribed by CbcScript II reverse transcriptase and 9-nt random primer. The products of reverse transcription were labeled with KLENOW polymerase and 9-nt random primer. Labeled cDNA with nucleotides coupled to a fluorescent dye (either Cy3 or Cy5) dCTP (GE Healthcare Cat. No. PA 55021/PA 53021) was hybridized to 22k human Genome Array Genechips (CapitalBio, Beijing, China) using two-channel microarray technology. LuxScan 3.0 (CapitalBio, Beijing, China) was used to analyze the image of microarray, and the two channel ratio values were normalized by the method of Lowess. Cy3/Cy5 ratios were log-transformed (base 2), median centered by arrays and genes, and hierarchically clustered (average linkage correlation metric). Gene products with at least 2-fold decreases (log2 ≤ –0.3) were determined as the significant differences of expression. Statistical comparisons were done by using the one class method in significance analysis of microarray (SAM) software 3.0 (Stanford University, USA).

### Protein extraction and western blot analysis

The total proteins were isolated from the HNSCC cell lines using RIPA buffer (50 mM Tris/HCl, pH 8.0, 250 mM NaCl, 1% Nonidet P-40, 0.5% (w/v) sodium deoxycholate, 0.1% sodium dodecylsulfate, Protease Inhibitor Cocktail Tablets (Roche, Mannheim, Germany)). Fifty micrograms of protein of different groups were boiled for 5 min in sample buffer and were then separated in sodium dodecyl sulphate–polyacrylamide gel electrophoresis and transferred on to a polyvinylidene fluoride membrane (Bio-Rad Laboratories, Hercules, CA, USA). Nonspecific reactivity was blocked using 5% bovine serum albumin in a TBST buffer (100 ml 10 × TBS, 10 ml 10% Tween20 final 0.1% v/v, dissolve with 890 ml double distilled-H2O) and then subsequently and probed with Human AREG MAb (R&D, Minneapolis, USA) overnight at 4°C. Membranes were also probed with anti-β-actin (Santa Cruz Biotechnology, Texas, USA) to assure equal amounts of protein. Bound antibodies were detected using horseradish peroxidase-conjugated secondary antibody (Santa Cruz Biotechnology, Texas, USA). Western blot experiments were repeated three times to confirm the results. The protein amounts were estimated through densitometry as the ratio detected protein/β-actin.

### Cloning of 3′UTRs

The full length of 3′UTRs of AREG mRNA containing putative miR-34a binding site was PCR-amplified using cDNA from Fadu cells. The 3′UTRs were cloned into the XhoI and NotI sites of the pSiCheck™-2 vector (Promega, Madison, WI, USA) and verified by sequencing. Seed matching sequences were mutated with the Quick Change Mutagenesis kit (Stratagene) using the AREG 3′UTR luciferase construct as a template. Oligonucleotides used for cloning and mutagenesis were provided as follows: AREG-3′UTR-xho I-F: CTCGAGGATATCACATTGGAGTCACTG; AREG-3′UTR-Not I-R: GCGGCCGCAGCTGTAAAATAAATACACTTC; AREG mut-F: GAGT***gtg***T***cgg***AAGTCATAGCCATAAATGATGAGTCG; AREG mut-R: CTATGACTT***ccg***A***cac***ACTCCAATGTGATATCCTG.

### Luciferase assays

For reporter assays, 5.0 × 10^4^ fadu and SCC-23 cells were co-transfected with 0.5 ug of the pSiCheck™-2 firefly luciferase vector (containing wild-type or mutated 3′UTR sequence), 50 ng Renilla luciferase control vector, and 30 pmol miR-34a or miR-control, using Lipofectamine 2000 (Invitrogen, Carlsad, CA). Lysates were collected 48 hr after transfection, and firefly luciferase activities were measured with a Dual-Luciferase Reporter System (Promega, Madison, WI, USA). Renilla luciferase was co-transfected and luminescence was measured to normalize transfection efficiency.

### Bioinformatic and statistical analysis

TargetScan5.2 (http://www.targetscan.org/) and Pict-Tar (http://pictar.mdc-berlin.de/) were used to predict the target genes of miR-34a. According to TargetScan, highly conserved predictions were included as the potential targets. All statistical analyses were performed using 13.0 SPSS software system. All data are presented as the mean ± SD; groups were compared using two tailed Student's *t*-test. *p* values < 0.01 were considered statistically significant.

The microarray data was deposited in the Gene expression omnibus (GEO) database. The following link has been created to allow reviewing: http://www.ncbi.nlm.nih.gov/geo/query/acc.cgi?token=spkteomuttuvnqd&acc=GSE59617.

## SUPPLEMENTARY FIGURES


