# Supplementary material for: MiR-34a suppresses amphiregulin and tumor metastatic potential of head and neck squamous cell carcinoma (HNSCC)
Source: Oncotarget. 2015 Feb 5;6(10):7454–69. doi: 10.18632/oncotarget.3148 (PMC4480692; doi:10.18632/oncotarget.3148)
Supplement: Supplementary file 1 [file oncotarget-06-7454-s001.pdf]

SUPPLEMENTARY FIGURES

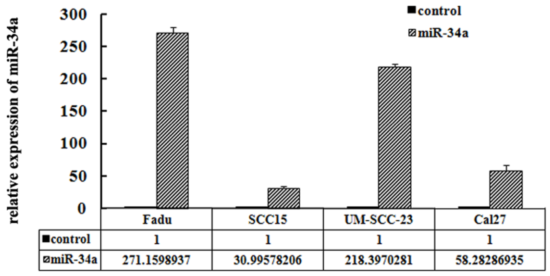

	Fadu	SCC15	UM-SCC-23	Cal27
control	1	1	1	1
miR-34a	271.1598937	30.99578206	218.3970281	58.28286935

Supplementary Figure 1: The relative miR-34a levels in miR-34a over-expression cells and the control cells.

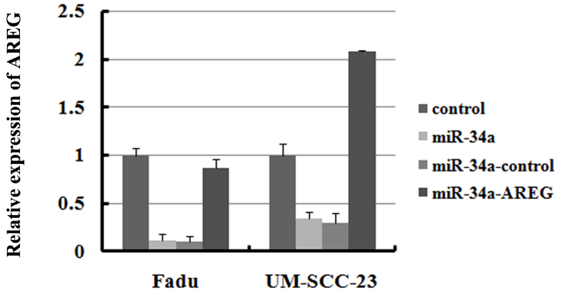

Supplementary Figure 2: The relative AREG mRNA levels in stable clones of Fadu-miR-34a-AREG and UM-SCC-23-miR-34a-AREG cells.

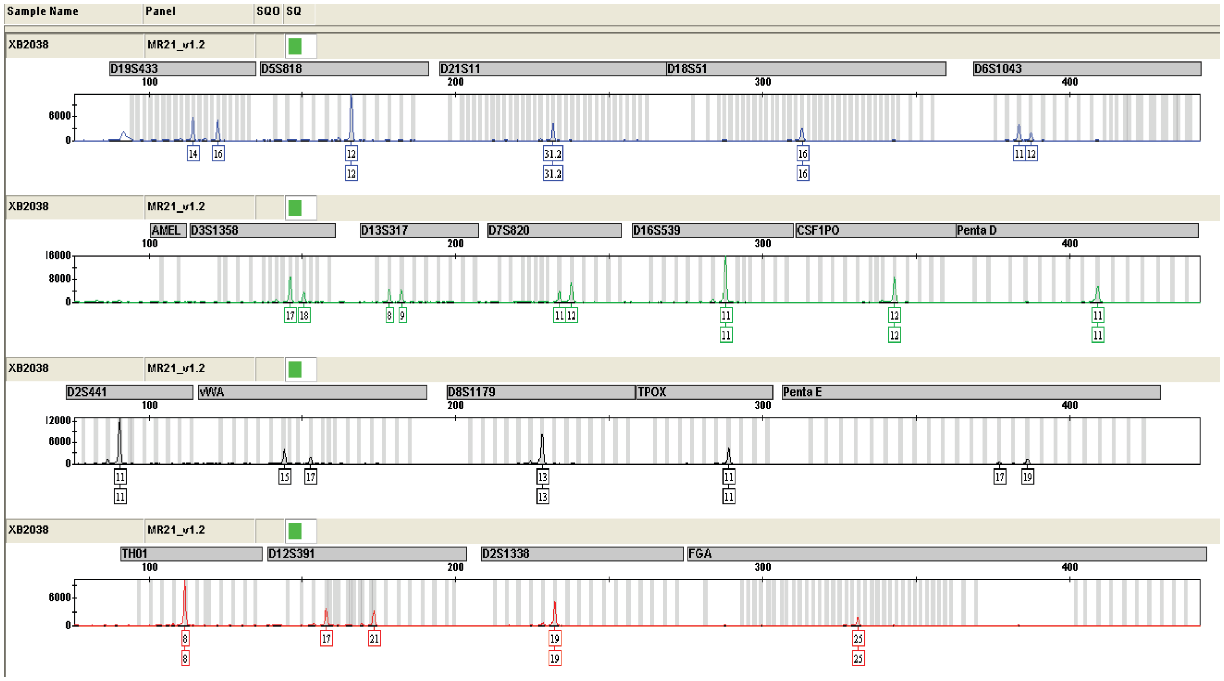

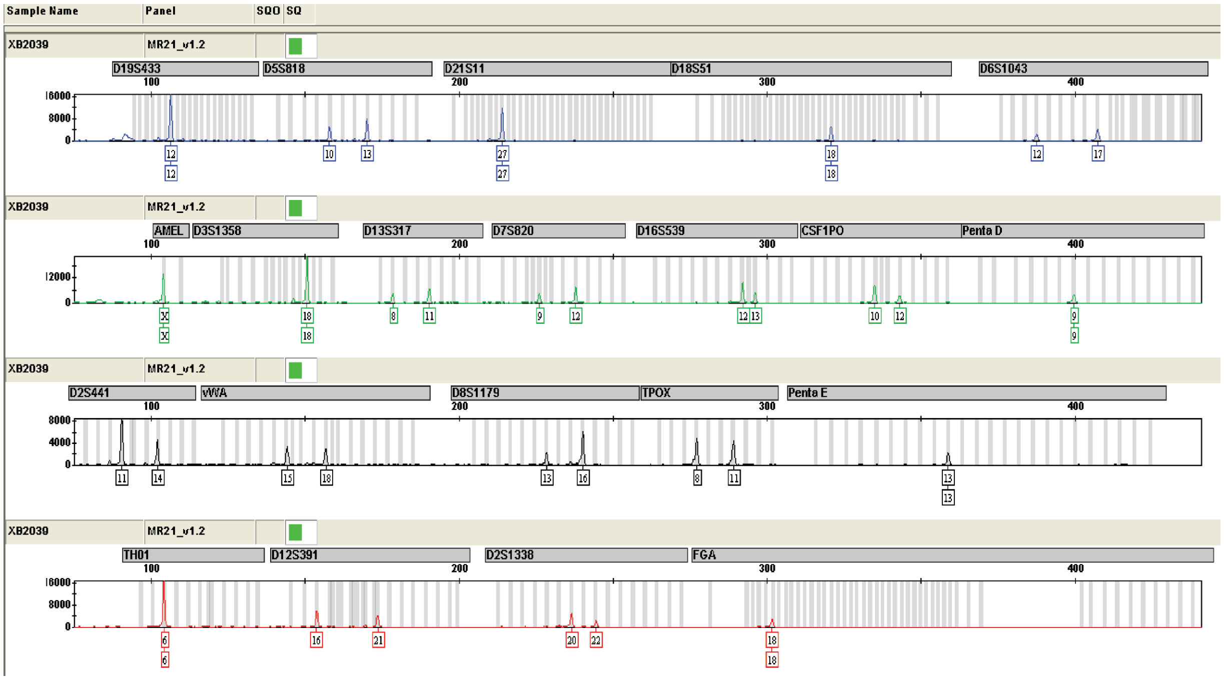

Supplementary Figure 3: The report of Fadu, UM-SCC-23, and Cal27 Cell Lines authentication. (*Continued*)

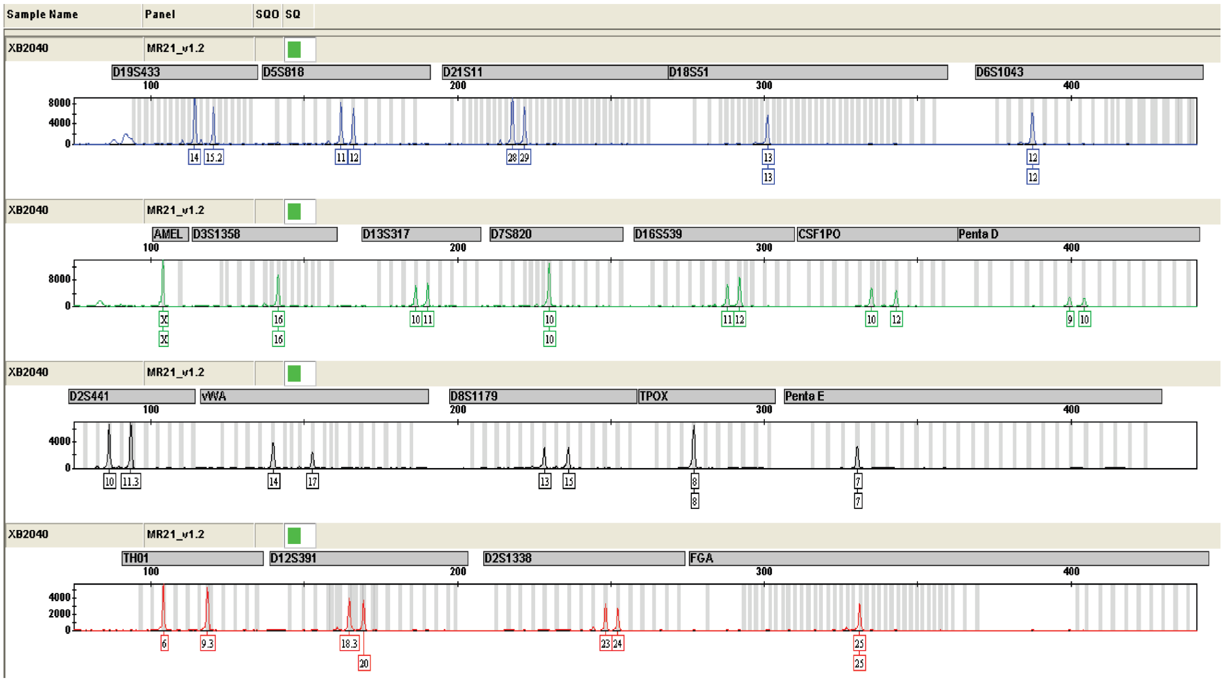

Supplementary Figure 3: (*Continued*) The report of Fadu, UM-SCC-23, and Cal27 Cell Lines authentication.
